# Assessing anemia burden and multifactorial contributors among pregnant women in Gujarat: a cross-sectional study integrating biochemical, nutritional, and geographic disparities

**DOI:** 10.3389/fgwh.2025.1717148

**Published:** 2025-12-12

**Authors:** Ashwini Agarwal, Harsh Bakshi, A. M. Kadri, Krupal Joshi, Astha Vala, Sagar Dholariya, Amit Sonagra, Manisha Upadhyay, Garima Anandani, Gyanendra Singh, Parth Goswami, Anemia Burden Assessment Group

**Affiliations:** 1 Department of Microbiology, All India Institute of Medical Sciences (AIIMS), Rajkot, Gujarat, India; 2 Department of Health and Family Welfare, State Health System Resource Centre (SHSRC), Gandhinagar, Gujarat, India; 3 Department of Community Medicine, GMERS Medical College Sola, Ahmedabad, Gujarat, India; 4 Department of Community and Family Medicine, All India Institute of Medical Sciences (AIIMS), Rajkot, Gujarat, India; 5 Department of Biochemistry, All India Institute of Medical Sciences (AIIMS), Rajkot, Gujarat, India; 6 Department of Pathology, All India Institute of Medical Sciences (AIIMS), Rajkot, Gujarat, India

**Keywords:** anemia, pregnancy, prevalence, micronutrients, biomarkers, dietary diversity

## Abstract

**Introduction:**

Anemia remains a critical public health challenge in India, particularly among pregnant women, where its multifactorial etiology is often underappreciated. Despite long-standing supplementation programs, anemia prevalence in Gujarat remains high, necessitating granular, region-specific investigations.

**Objectives:**

To assess the prevalence of anemia among pregnant women across ten districts of Gujarat, and to identify key sociodemographic, nutritional, hematological, and biochemical determinants contributing to anemia and its geographic disparities.

**Methodology:**

This community-based study included 2,805 pregnant women from diverse settings (tribal/rural/urban). Hematological and biochemical assessments included serum ferritin, iron, C-Reactive Protein (CRP), folate, vitamin B12, prealbumin, and hemoglobinopathy screening. A logistic regression analysis was conducted to determine the independent factors associated with anemia, with the findings presented as adjusted odds ratios (aOR) along with their 95% confidence intervals (CI).

**Results:**

Overall anemia prevalence was 64.2%, with mild anemia comprising 82.1% of cases. Tribal women had 2.21-fold higher odds of anemia than urban counterparts (aOR = 2.21, 95% CI: 1.88–2.61, *p* < 0.001). Anemia was also associated with illiteracy (aOR = 2.16, *p* < 0.001), underweight status (aOR = 1.58, *p* < 0.001), and low dietary diversity (aOR = 2.26, *p* < 0.001). Biochemical assessments revealed absolute iron deficiency in 17.2%, folate deficiency in 15.5%, and vitamin B12 deficiency in 60.3% of anemic women. Elevated CRP levels indicated inflammation in 34.7%. Multivariable binary logistic regression revealed five significant and independent predictors of anemia: reduced red blood cell count (aOR = 0.26; 95% CI: 0.22–0.31), elevated red cell distribution width (RDW-CV) (aOR = 1.39; 95% CI: 1.33–1.46), diminished serum prealbumin (aOR = 0.92; *p* < 0.001), lower folate levels (aOR = 0.97; *p* < 0.001), and decreased ferritin concentrations (aOR = 0.99; *p* < 0.001), each independently contributing to anemia risk.

**Conclusion:**

Anemia in Gujarat's pregnant women is highly prevalent and multifactorial, shaped by overlapping nutritional, inflammatory, and social determinants. Region-specific, integrated strategies targeting biochemical deficits and structural inequities are essential for effective anemia mitigation.

## 1 Introduction

Anemia in pregnancy remains a major global public health challenge, contributing significantly to maternal and neonatal morbidity and mortality. According to the World Health Organization (WHO), approximately 37% of pregnant women worldwide equivalent to more than 32 million women are anemic. The WHO Regions of Africa and South-East Asia are most affected with an estimated 106 million women in Africa and 244 million women in South-East Asia (1). Despite substantial global progress in maternal health, anemia continues to be responsible for adverse pregnancy outcomes such as preterm birth, low birth weight, intrauterine growth restriction, and increased maternal mortality (2, 3). The WHO has identified the reduction of anemia among women of reproductive age as a key global nutrition target for 2025, underscoring its importance in achieving the Sustainable Development Goals (4).

In India, the challenge is particularly acute. Despite decades of concerted public health interventions, the prevalence of anemia among pregnant women has remained high. The National Family Health Survey-5 (NFHS-5, 2019–21) reported that 52.2% of pregnant women in India were anemic, representing only marginal improvement from earlier rounds (5). Despite the efforts of programs like the National Iron Plus Initiative (NIPI), Anemia Mukt Bharat (AMB), and POSHAN Abhiyaan in scaling up iron and folic acid (IFA) supplementation and promoting nutritional awareness, progress has been inconsistent and remains inadequate across different states and regions (6–8). These initiatives have focused primarily on iron and folic acid (IFA) supplementation, deworming, and nutrition education, yet they have not adequately addressed the multifactorial etiology of anemia.

Recent research emphasizes that anemia in pregnancy is not solely due to iron deficiency (9). Non-iron deficiency anemia (NIDA), attributed to vitamin B12 and folate deficiencies, chronic inflammation, infection, and protein-energy malnutrition, is emerging as a significant and underrecognized contributor (9).

In India, where large sections of the population follow predominantly vegetarian diets and have limited dietary diversity, the risk of multiple micronutrient deficiencies is high. Additionally, structural and social determinants including education, caste, sanitation, and food insecurity play a critical role in shaping maternal nutrition and anemia outcomes (10, 11).

Within this national context, Gujarat presents a compelling case for focused investigation. Despite being one of India's economically progressive states, Gujarat continues to report a high prevalence of anemia among pregnant women (62.6%) according to NFHS-5—which is notably higher than the national average (12). More importantly, marked inter-district disparities exist across the state. For instance, according to NFHS-5, anemia prevalence among pregnant women ranges from over 70% in several tribal districts (such as Dahod and Narmada) to below 50% in urbanized districts (like Ahmedabad and Surat). These variations reflect the diverse socio-demographic, cultural, and geographic context of Gujarat—comprising tribal, rural, and urban regions—each with distinct dietary practices, food availability, sanitation standards, and healthcare accessibility (13). Further, tribal and rural populations in Gujarat often face higher levels of undernutrition, limited access to quality antenatal care, and lower levels of nutrition literacy, contributing to their disproportionate anemia burden (13). Despite these differences, most national and state programs adopt uniform anemia control strategies, potentially overlooking the local determinants and inter-district variations in the causes of anemia. This context establishes a clear rationale for selecting Gujarat as the study area. Its socioeconomic advancement juxtaposed with persistently high and uneven anemia prevalence provides a unique opportunity to explore the complex interplay of nutritional, biochemical, and sociodemographic factors that influence maternal health. Understanding these regional nuances is critical for designing context-specific, evidence-driven interventions that can enhance the effectiveness of ongoing national initiatives such as Anemia Mukt Bharat.

Although several studies in India have reported the prevalence and determinants of anemia among pregnant women, most have primarily focused on dietary intake or sociodemographic correlates, with limited integration of biochemical parameters and geographic variations. Evidence from Gujarat, in particular, remains fragmented, with few comprehensive analyses linking nutritional deficiencies (iron, vitamin B12, and folate) to sociodemographic and regional disparities. This gap limits the ability to design targeted interventions responsive to local needs. Hence, the present study was designed to provide a comprehensive, multidimensional profile of anemia and malnutrition among pregnant women across ten purposively selected districts of Gujarat, representing urban, rural, and tribal populations. The present study therefore addresses this evidence gap by adopting a multidimensional approach—integrating biochemical, nutritional, and geographic analyses—to generate context-specific insights that can inform tailored strategies under national initiatives such as Anemia Mukt Bharat and the Reproductive, Maternal, Newborn, Child, and Adolescent Health (RMNCHA + N) framework.

So, this study sought to estimate the prevalence of anemia and malnutrition among pregnant women across ten purposively selected districts in Gujarat. It further sought to identify key sociodemographic, nutritional, and biochemical determinants contributing to these conditions. Additionally, the study analyzed geographic disparities in anemia burden, with attention to differences across rural, urban, and tribal populations.

## 2 Methodology

### 2.1 Study design and setting

The present study, conducted from May 2024 to April 2025, employed a community-based cross-sectional design to investigate the prevalence and determinants of anemia among pregnant women across ten districts in Gujarat, India—namely Anand, Dahod, Jamnagar, Mehsana, Navsari, Rajkot, Sabarkantha, Surat, Tapi, and Vadodara. A stratified multistage sampling approach was adopted, with districts categorized based on their rural, urban, and tribal composition. Within each stratum, Primary Health Centers (PHCs) were selected using Probability Proportional to Size (PPS) sampling, based on the population served. From the selected PHCs, eligible participants were randomly recruited from antenatal care registries and adolescent health records.

### 2.2 Sample size determination

To ensure robust prevalence estimates, we powered our study to detect anemia rates with 8% precision at 95% confidence using formula DEFF*Np(1-p)]/ [(d2/Z21-α/2*(N-1)+p*(1-p). Drawing from previous reports showing 62.2% prevalence and accounting for our multi-stage sampling design (design effect = 2), we enrolled 250–280 women per district, culminating in 2,805 participants across all study sites. The choice of an 8% absolute precision was based on established methodological guidance for community-based prevalence surveys, where a precision range of 5%–10% is considered acceptable for estimating common conditions such as anemia in large populations. Similar precision parameters have been adopted in national surveys, including the National Family Health Survey (NFHS) and district-level nutrition assessments.

### 2.3 Inclusion and exclusion criteria

The study comprised pregnant women in any trimester who had resided in the respective districts for at least six months. Eligible participants were required to provide written informed consent. Inclusion extended to those currently on iron or micronutrient supplementation to evaluate the impact of ongoing interventions. Exclusion criteria encompassed individuals with severe pregnancy complications requiring emergency care, recent migrants (residing in the district for less than six months), and those unwilling to participate in dietary or biochemical assessments. Additionally, participants with chronic illnesses known to significantly alter hematological parameters—such as uncontrolled diabetes, advanced renal disease, tuberculosis under treatment, or malignancy—were excluded.

### 2.4 Data collection method

Data were gathered through direct, in-person interviews using a structured and pre-tested questionnaire. The tool collected details on participants’ demographics, dietary habits, medical history, access to sanitation, and use of antenatal and nutritional services. Anthropometric measurements, including weight and height, were recorded using standardized procedures, and body mass index (BMI) was determined accordingly. Anemia was defined according to World Health Organization (WHO) criteria for pregnant women with hemoglobin levels <11.0 g/dL. Severity was further stratified as mild, moderate, or severe based on WHO thresholds (14).

### 2.5 Biochemical and hematological analysis

Biochemical data were obtained through venous blood samples (6–8 mL), collected under aseptic conditions into both plain and Ethylenediaminetetraacetic acid (EDTA) vacutainers (15). All laboratory analyses were conducted using standardized instruments and protocols (16). Hematological parameters were measured using the Sysmex XN-1000 six-part hematology analyzer (17), while biochemical assays were performed using the VITROS 5600 platform (18), employing dry chemistry and enhanced electrochemiluminescence methods. In addition to hemoglobin, a broad panel of indicators was evaluated, including hematological parameters, serum iron, total iron-binding capacity (TIBC), transferrin, and serum ferritin. Vitamin status was assessed by serum vitamin B12 and folate levels. Other nutritional markers included serum albumin, prealbumin, magnesium, and vitamin D. C-reactive protein (CRP) was measured as a indicator of inflammation, and hemoglobin variants were identified where indicated. Peripheral blood smears were prepared using Leishman and field staining techniques for morphological examination (19).

### 2.6 Ethical considerations

The study received ethical approval from the Institutional Ethics Committee of AIIMS Rajkot (Approval No: AIIMS/RAJKOT/5th IEC/FB/37). All participants were informed about the purpose of the study, the voluntary nature of participation, potential risks and benefits, and their right to withdraw at any time without consequences. Written informed consent was obtained prior to enrolment; for participants with limited literacy, the consent form was read aloud in their local language and a thumb impression was obtained in the presence of an impartial witness.

Confidentiality of data was ensured through de-identification of participant records, secure storage of digital data in password-protected systems, and restricted access to study personnel only. No personal identifiers were included in the analysis or dissemination of findings.

Given that the study involved pregnant women—a group considered vulnerable—additional safeguards were employed. Interviewers were trained to conduct data collection in a private setting, ensure psychological comfort, and minimize respondent burden. Only non-invasive procedures were used, and participation had no implications for clinical care or eligibility for health services. These measures ensured adherence to national ethical guidelines for research involving vulnerable populations.

### 2.7 Statistical analysis

Data were analyzed using Microsoft Excel 2010 and Jamovi (version 2.5.3.0). Descriptive statistics were applied to summarize the data—means with standard deviations for continuous variables and proportions for categorical variables. Associations between anemia status and other variables were assessed using chi-square tests for categorical data. Continuous variables were compared between anemic and non-anemic groups using independent *t*-tests for normally distributed data and Mann–Whitney *U*-tests for non-normally distributed data. For our analysis, we classified participants as either anemic or non-anemic using the World Health Organization's established hemoglobin cutoffs for pregnancy. We examined several potential influencing factors across different categories: personal characteristics like age, education level, and social background; nutritional patterns measured through dietary diversity scores; and laboratory results including iron stores (ferritin), vitamin B12 and folate levels, and markers of inflammation (CRP). The strength of each factor's relationship with anemia risk was calculated and presented as adjusted odds ratios along with their 95% confidence ranges.

To validate the suitability of our final logistic regression model, we planned to assess its goodness-of-fit using the Hosmer-Lemeshow test and evaluate the potential for multicollinearity among the included variables using the Variance Inflation Factor (VIF). Statistical significance was determined using a threshold of *p* < 0.05.”

## 3 Results

### 3.1 Demographic profile and anemia prevalence among study participants

**Figure 1:**
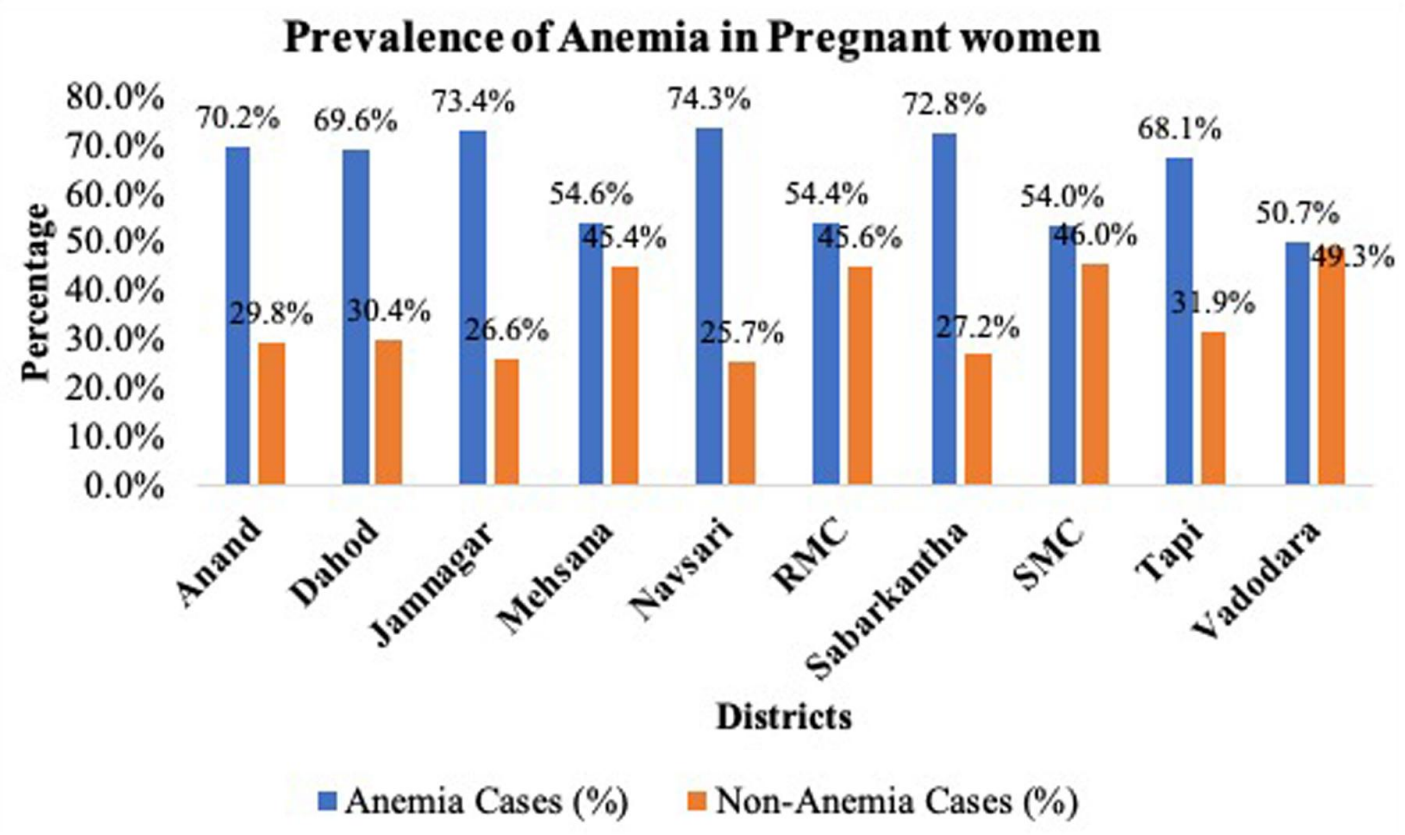
District wise prevalence of anemia among pregnant women.

A total of 2,805 pregnant women were included in the study. Most belonged to tribal (40.5%), followed by urban (30.2%) and rural (29.3%) areas. The majority were aged 20–24 years (46.0%), had received primary (30.1%) or secondary (29.2%) education, and lived in pukka houses (57.4%). Nearly 50.8% of participants were from below-poverty-line households, and 37.8% had health insurance. The majority were in their second (43.7%) or third (39.9%) trimester. The prevalence of anemia was 64.2%, of which 82.1% had mild, 13.9% moderate, and 2.7% severe anemia (Table 1). Figure 1 illustrates the district-wise prevalence of anemia among pregnant women, with the highest prevalence observed in Navsari district, followed by Jamnagar and sabarkantha district.

**Table 1:** Baseline characteristics, prevalence, and severity of anemia in pregnant women.

Variables	Subgroup	Pregnant women (*N* = 2,805)
Location and socio-cultural classification	Urban	847 (30.2%)
	Rural	821 (29.3%)
	Tribal	1,137 (40.5%)
Age	15–19 years	194 (6.9%)
	20–24 years	1,291 (46.0%)
	25–29 years	879 (31.3%)
	30–34 years	361 (12.9%)
	35–39 years	72 (2.6%)
	> 40 years	8 (0.3%)
Education level	Unknown	11 (0.4%)
	Primary education (up to 8th)	844 (30.1%)
	Secondary education (9th–10th)	820 (29.2%)
	Higher secondary education (11th–12th)	416 (14.8%)
	Graduate/college	258 (9.2%)
	Master's degree	49 (1.7%)
	No formal education but can read and write	75 (2.7%)
	No formal education and cannot read and write	332 (11.8%)
Type of houses	Pukka	1,609 (57.4%)
	Kutcha	591 (21.1%)
	Semi-pukka	605 (21.6%)
Type of family	Nuclear	610 (21.5%)
	Extended	960 (34.2%)
	Joint	1,235 (44.0%)
Religion	Christianity	53 (1.9%)
	Hinduism	2,523 (89.9%)
	Islam	229 (8.2%)
Caste	Don't know or no response	533 (19.0%)
	General	398 (14.2%)
	OBC	779 (27.8%)
	SC	262 (9.3%)
	ST	833 (29.7%)
Family with below poverty line card	Don't know or no response	228 (8.1%)
	No	1,153 (41.1%)
	Yes	1,424 (50.8%)
Health insurance coverage	Don't know or no response	293 (10.4%)
	No	1,452 (51.8%)
	Yes	1,060 (37.8%)
Using mobile/computer/laptop/tablet	Yes	2,527 (90.2%)
	No	279 (9.8%)
Pregnancy trimester	1st Trimester (1–3 months)	461 (16.4%)
	2nd Trimester (4–6 months)	1,226 (43.7%)
	3rd Trimester (7–9 months)	1,118 (39.9%)
Prevalence of anemia	Anemic	1,801 (64.2%)
	Non-anemic	1,004 (35.8%)
Severity of anemia	Mild anemia (Hb 9–10.9 g/dL)	1,498 (82.1%)
	Moderate anemia (Hb 7–8.9 g/dL)	254 (13.9%)
	Severe anemia (Hb <7 g/dL)	49 (2.7%)

### 3.2 Prevalence of anemia by sociodemographic and obstetric factors

The prevalence of anemia was highest among tribal women (71.2%), followed by rural (66.0%), and urban women (53.0%). Women who belong to tribal areas are 2.21-fold more likely to have anemia than their urban counterparts (aOR = 2.21, 95% CI: 1.88–2.61, *p* < 0.001). Higher anemia prevalence was also observed in scheduled tribes (72.3%, aOR 2.07; 95% CI: 1.61–2.66; *p* < 0.001), kutcha (69.0%, aOR 1.40; 95% CI: 1.14–1.72; *p* = 0.001) and semi-pukka houses (66.9%, aOR 1.27; 95% CI: 1.04–1.55; *p* = 0.018), and among women with no health insurance (61.9%, aOR 0.82; 95% CI: 0.68–0.98; *p* = 0.031).

**Table 2:** Association of sociodemographic, obstetric, and healthcare-related determinants with prevalence of anemia.

Variable	Category	Anemic (*n* = 1,801)	Non-anemic (*n* = 1,004)	aOR (95% CI)	*p*-value
Socio-demographic characteristics					
Location	Urban (ref)	449 (53.0%)	398 (47.0%)	1.00	**<0** **.** **001**
	Rural	542 (66.0%)	279 (34.0%)	**1.73 (1**.**42–2.11)**	**<0** **.** **001**
	Tribal	810 (71.2%)	327 (28.8%)	**2.21 (1**.**83–2.67)**	**<0** **.** **001**
Caste	General (ref)	223 (56.0%)	175 (44.0%)	1.00	**<0** **.** **001**
	OBC	456 (58.5%)	323 (41.5%)	1.11 (0.87–1.42)	0.396
	SC	170 (64.9%)	92 (35.1%)	**1.45 (1**.**05–2.00)**	**0** **.** **024**
	ST	602 (72.3%)	231 (27.7%)	**2.07 (1**.**61–2.66)**	**<0** **.** **001**
House type	Pukka (ref)	988 (61.4%)	621 (38.6%)	1.00	**0** **.** **001**
	Kutcha	408 (69.0%)	183 (31.0%)	**1.40 (1**.**14–1.72)**	**0** **.** **001**
	Semi-pukka	405 (66.9%)	200 (33.1%)	**1.27 (1**.**04–1.55)**	**0** **.** **018**
Below poverty line	No (ref)	711 (61.7%)	442 (38.3%)	1.00	0.072
	Yes	928 (65.2%)	496 (34.8%)	1.16 (0.99–1.36)	0.069
Healthcare access and utilization					
Place of last delivery	Government (ref)	561 (69.7%)	244 (30.3%)	1.00	**0** **.** **024**
	Private	336 (59.8%)	226 (40.2%)	**0.65 (0**.**51–0.81)**	**<0** **.** **001**
	Home/traditional	35 (81.4%)	8 (18.6%)	**1.90 (0**.**86–4.20)**	0.112
Health insurance	Yes (ref)	704 (66.4%)	356 (33.6%)	1.00	**0** **.** **023**
	No	899 (61.9%)	553 (38.1%)	**0.82 (0**.**68–0.98)**	**0** **.** **031**
Healthcare utilization					
Mamta card possession	No (ref)	41 (59.4%)	28 (40.6%)	1.00	0.401
	Yes	1,760 (64.3%)	976 (35.7%)	1.22 (0.77–1.94)	0.405
Attended Mamta Divas	No (ref)	310 (61.3%)	196 (38.7%)	1.00	0.127
	Yes	1,491 (64.8%)	808 (35.2%)	1.16 (0.96–1.40)	0.130
Anganwadi services	No (ref)	494 (58.3%)	354 (41.7%)	1.00	**<0** **.** **001**
	Yes	1,307 (66.8%)	650 (33.2%)	**1.43 (1**.**23–1.66)**	**<0** **.** **001**
Government monetary benefits	No (ref)	1,424 (64.0%)	802 (36.0%)	1.00	0.610
	Yes	377 (65.1%)	202 (34.9%)	1.05 (0.87–1.26)	0.615
Obstetric and reproductive history					
Parity	Nulliparous (ref)	827 (63.0%)	485 (37.0%)	1.00	0.444
	1–2 pregnancies	833 (65.4%)	440 (34.6%)	1.11 (0.94–1.31)	0.237
	>2 pregnancies	141 (64.1%)	79 (35.9%)	1.05 (0.77–1.43)	0.760
Previous adverse outcome	No (ref)	741 (66.5%)	372 (33.5%)	1.00	0.123
	Yes	235 (62.0%)	145 (38.0%)	0.82 (0.64–1.06)	0.132
Age at first pregnancy	>21 years (ref)	425 (61.2%)	269 (38.8%)	1.00	0.002
	≤21 years	549 (68.7%)	250 (31.3%)	1.42 (1.14–1.77)	0.002
Interpregnancy interval	≥24 months (ref)	473 (65.4%)	250 (34.6%)	1.00	0.972
	<24 months	507 (65.4%)	269 (34.6%)	1.00 (0.81–1.24)	0.972
Current pregnancy details					
Trimester	1st (ref)	241 (52.3%)	220 (47.7%)	1.00	**<0** **.** **001**
	2nd	865 (76.9%)	361 (23.1%)	**3.04 (2**.**44–3.78)**	**<0** **.** **001**
	3rd	695 (62.2%)	423 (37.8%)	**1.50 (1**.**20–1.87)**	**<0** **.** **001**
High-risk pregnancy	No (ref)	1,328 (62.5%)	799 (37.5%)	1.00	**0** **.** **001**
	Yes	402 (68.8%)	182 (31.2%)	**1.33 (1**.**09–1.62)**	**0** **.** **005**
Pregnancy registration	Yes (ref)	1,741 (64.6%)	953 (35.4%)	1.00	**0** **.** **023**
	No	60 (54.1%)	51 (45.9%)	**0.64 (0**.**43–0.95)**	**0** **.** **028**

Associations were assessed using chi-square tests for bivariate analyses. Adjusted odds ratios (aORs) and 95% confidence intervals (CIs) were derived from multivariable logistic regression, with *p*-values calculated via Wald tests for individual coefficients. Bold text represents significant *p*-value (*p* < 0.05).

Pregnant women with high-risk pregnancies (68.8%, aOR 1.33; 95% CI: 1.09–1.62; *p* = 0.005), those with age at first pregnancy ≤21 years (68.7%, aOR 1.42; 95% CI: 1.14–1.77; *p* = 0.002), and those in their second trimester (76.9%, aOR 3.04; 95% CI: 2.44–3.78; *p* < 0.001) had higher anemia prevalence. The odds were also significantly higher among women who did not register their pregnancy (54.1% vs. 64.6%, aOR 0.64; 95% CI: 0.43–0.95; *p* = 0.028) (Table 2).

### 3.3 Medical and nutritional factors associated with anemia

Participants with sickle cell/thalassemia (72.3%, aOR 1.48; 95% CI: 1.01–2.18; *p* = 0.045) and a history of previous anemia (71.4%, aOR 1.45; 95% CI: 1.13–1.86; *p* = 0.004) showed higher anemia prevalence. Those who consumed IFA supplements during pregnancy had slightly higher anemia prevalence (64.9%) compared to those who did not (59.3%), with significant association for IFA intake (aOR 1.27; 95% CI: 1.01–1.59; *p* = 0.040). Twice daily IFA intake showed a stronger association (aOR 1.62; 95% CI: 1.28–2.05; *p* < 0.001), and high-frequency IFA intake (≥4 days/week) was also associated with increased odds of anemia (aOR 1.93; 95% CI: 1.01–3.68; *p* = 0.046). No significant differences were observed by IFA side effects or method of IFA intake (Table 3).

**Table 3:** Association of medical/nutritional intervention and IFA supplementation factors with prevalence of anemia.

Variable	Category	Anemic (*n*, %)	Non-anemic (*n*, %)	aOR (95% CI)	*p*-value
Medical interventions					
Intravenous injection in current pregnancy	No (ref)	1,416 (63.7%)	809 (36.3%)	1.00	0.311
	Yes	353 (66.0%)	182 (34.0%)	1.10 (0.91–1.34)	0.315
Blood transfusion history	No (ref)	1,703 (63.9%)	964 (36.1%)	1.00	0.073
	Yes	91 (71.7%)	36 (28.3%)	1.42 (0.96–2.10)	0.078
Sickle cell/thalassemia	No (ref)	1,515 (63.8%)	860 (36.2%)	1.00	**0** **.** **043**
	Yes	73 (72.3%)	28 (27.7%)	**1.48 (1** **.** **01–2.18)**	**0** **.** **045**
Worm infestation	No (ref)	1,666 (63.7%)	953 (36.3%)	1.00	0.627
	Yes	23 (67.7%)	11 (32.3%)	1.18 (0.60–2.32)	0.630
Pica (non-food items)	No (ref)	1,719 (64.0%)	970 (36.0%)	1.00	0.137
	Yes	82 (70.7%)	34 (29.3%)	1.32 (0.91–1.91)	0.140
Anemia history					
Previous anemia	No (ref)	709 (63.3%)	412 (36.7%)	1.00	**0** **.** **004**
	Yes	220 (71.4%)	86 (28.6%)	**1.45 (1** **.** **13–1.86)**	**0** **.** **004**
Anemia testing (current pregnancy)	No (ref)	625 (62.3%)	378 (37.7%)	1.00	0.119
	Yes	1,176 (65.3%)	626 (34.7%)	1.13 (0.97–1.32)	0.122
IFA supplementation					
IFA consumption (current pregnancy)	No (ref)	208 (59.3%)	143 (40.7%)	1.00	**0** **.** **039**
	Yes	1,593 (64.9%)	861 (35.1%)	**1.27 (1** **.** **01–1.59)**	**0** **.** **040**
IFA frequency (last month)	Did not consume (Ref)	216 (59.0%)	150 (41.0%)	1.00	**<0** **.** **001**
	Occasionally	6 (66.7%)	3 (33.3%)	1.38 (0.34–5.58)	0.650
	3 days/week	15 (71.4%)	6 (28.6%)	1.72 (0.66–4.50)	0.270
	≥4 days/week	37 (74.0%)	13 (26.0%)	**1.93 (1** **.** **01–3.68)**	**0** **.** **046**
	Once daily	824 (60.7%)	533 (39.3%)	1.07 (0.86–1.34)	0.540
	Twice daily	703 (70.2%)	299 (29.8%)	**1.62 (1** **.** **28–2.05)**	**<0** **.** **001**
IFA consumption method	With meal + water (ref)	1,243 (65.5%)	655 (34.5%)	1.00	0.390
	With meal + lemon	88 (59.9%)	59 (40.1%)	0.79 (0.57–1.10)	0.170
	With meal + milk/tea	54 (65.1%)	29 (34.9%)	0.98 (0.62–1.55)	0.940
	Before meal + water	189 (65.4%)	100 (34.6%)	0.99 (0.77–1.27)	0.930
IFA side effects	No (ref)	1,378 (64.8%)	748 (35.2%)	1.00	0.804
	Yes	215 (65.6%)	113 (34.4%)	1.03 (0.81–1.31)	0.810

Associations were assessed using chi-square tests for bivariate analyses. Adjusted odds ratios (aORs) and 95% confidence intervals (CIs) were derived from multivariable logistic regression, with *p*-values calculated via Wald tests for individual coefficients. Bold text represents significant p-value (*p* < 0.05).

### 3.4 Anemia knowledge and awareness

Lower anemia prevalence was significantly associated with higher knowledge scores. Participants with high knowledge (score 7–9) had the lowest anemia prevalence (18.5%), with significantly lesser odds compared to low knowledge scores (aOR 0.12; 95% CI: 0.09–0.15; *p* < 0.001). Moderate knowledge (60.8%) was also associated with lower odds (aOR 0.79; 95% CI: 0.66–0.95; *p* = 0.012) (Table 4).

**Table 4:** Association between anemia knowledge scores and anemia prevalence in pregnant women.

Overall knowledge (education score) regarding anemia	Anemic (*n*, %)	Non-anemic (*n*, %)	aOR (95% CI)	*p*-value (overall)
Low (0–3) (ref)	1,101 (66.3%)	559 (33.7%)	1.00	**<0** **.** **001**
Moderate (4–6)	394 (60.8%)	254 (0.66–0.95)	**0** **.** **012**	
High (7–9)	92 (18.5%)	405 (81.5%)	0.12 (0.09–0.15)	**<0** **.** **001**

The overall knowledge score was calculated based on nine binary (Yes/No) questions assessing awareness and understanding of anemia: (1) “Have you heard of anemia?” (2) “Do you know what causes anemia?” (3) “Do you know common symptoms?” (4) “Do you know prevention/treatment methods?” (5) “Are you aware of iron's dietary importance?” (6) “Can you list iron's roles in the body?” (7) “Do you know iron-rich foods?” (8) “Are you aware of IFA tablets?” and (9) “What is IFA's importance?” Each “Yes”/correct answer received 1 point (max score = 9), categorized as Low (0–3), Moderate (4–6), or High (7–9) knowledge. The score demonstrated good internal consistency (Cronbach's α = 0.78) and was adjusted for education/socioeconomic status in analyses. Bold values represents significant *p*-value.

### 3.5 Dietary, hygiene, and household conditions

Anemia prevalence was higher in participants with low dietary diversity scores (64.4%, aOR 2.26; 95% CI: 1.18–4.33; *p* = 0.014) related to particiapnts with high diversity (44.4%). Moderate FDS also showed increased odds (aOR 2.25; 95% CI: 1.16–4.37; *p* = 0.016). Those with household food insecurity had significantly higher anemia prevalence (75.6%, aOR 1.81; 95% CI: 1.40–2.34; *p* < 0.001). Use of communal toilets was also associated with higher anemia prevalence (70.7%, aOR 1.34; 95% CI: 1.05–1.71; *p* = 0.020) compared to private ones (Table 5).

**Table 5:** Association of dietary, hygiene, and household factors with prevalence anemia in pregnant women.

Variable	Category	Anemic (*n*, %)	Non-anemic (*n*, %)	aOR (95% CI)	*p*-value
Dietary habits					
Type of diet	Vegetarian (ref)	1,636 (64.3%)	908 (35.7%)	1.00	0.41
	Non-vegetarian	165 (61.6%)	103 (38.4%)	0.88 (0.69–1.13)	0.32
Tea/coffee consumption	None (ref)	286 (62.0%)	175 (38.0%)	1.00	0.643
	Tea	1,453 (64.5%)	800 (35.5%)	1.11 (0.90–1.37)	0.330
	Coffee	20 (66.7%)	10 (33.3%)	1.23 (0.57–2.64)	0.590
	Both tea & coffee	42 (68.9%)	19 (31.1%)	1.36 (0.77–2.41)	0.290
Food Diversity Score (FDS)	High (≥11) (ref)	16 (44.4%)	20 (55.6%)	1.00	**0** **.** **044**
	Moderate (6–10)	547 (64.6%)	300 (35.4%)	**2.25 (1** **.** **16–4.37)**	**0** **.** **016**
	Low (≤5)	1,238 (64.4%)	684 (35.6%)	**2.26 (1** **.** **18–4.33)**	**0** **.** **014**
Hygiene & sanitation					
Food preparation	Self (ref)	1,517 (64.8%)	824 (35.2%)	1.00	0.192
	Family members	283 (61.1%)	180 (38.9%)	0.86 (0.69–1.06)	0.160
Wash vegetables/fruits	Yes (ref)	1,778 (64.1%)	998 (35.9%)	1.00	0.161
	No	23 (79.3%)	6 (20.7%)	2.09 (0.85–5.15)	0.110
Type of toilet	Personal (ref)	831 (64.4%)	459 (35.6%)	1.00	**0** **.** **011**
	Communal	282 (70.7%)	117 (29.3%)	**1.34 (1** **.** **05–1.71)**	**0** **.** **020**
Household food security					
In the last month, did you worry that your Household would not have enough food	No (ref)	1,504 (63.0%)	883 (37.0%)	1.00	**0** **.** **001**
	Yes	279 (75.6%)	90 (24.4%)	**1.81 (1** **.** **40–2.34)**	**<0** **.** **001**

Bold values represents significant *p*-value.

### 3.6 Comparison of hematological and biochemical parameters

Anemic participants had significantly lower body weight, BMI, blood pressure, and RBC count than non-anemic individuals. Hematological parameters such as MCV, MCH, MCHC, HCT, and ferritin were all significantly lower in anemic participants, while RDW-CV, TIBC, and reticulocyte count were higher. Biochemical parameters showed lower serum iron (61.00 μg/dL vs. 86.00 μg/dL), ferritin (11.20 ng/mL vs. 17.30 ng/mL), folate (7.37 ng/mL vs. 11.20 ng/mL), vitamin B12 (212.00 pg/mL vs. 222.00 pg/mL), albumin (3.68 ± 0.87 g/dL vs. 3.81 ± 0.41 g/dL), and prealbumin (18.45 ± 4.40 mg/dL vs. 19.96 ± 4.74 mg/dL) in anemic individuals. Transferrin levels were higher in the anemic group (384.42 ± 89.01 mg/dL vs. 351.35 ± 69.95 mg/dL), and transferrin saturation was lower (13.01% vs. 20.10%) (Table 6).

**Table 6:** Comparison of demographics, anthropometrics, clinical, hematological, and biochemical parameters between anemic and non-anemic pregnant individuals.

Parameter	Anemia mean ± SD or median (IQR)* [*n* = 1,801]	Non-anemia mean ± SD or median (IQR)* [*n* = 1,004]	*P* value
Age (years)	24.7 ± 4.1	25.0 ± 4.2	0.06
Height (m)	1.5 ± 0.1	1.5 ± 0.1	0.48
Weight (kg)	50.9 ± 11.1	54.4 ± 13.4	<0.001
BMI (kg/m^2^)	22.2 ± 5.1	23.7 ± 6.2	<0.001
SBP (mmHg)	109.7 ± 12.1	111.2 ± 12.5	0.002
DBP (mmHg)	69.8 ± 8.9	71.2 ± 9.2	<0.001
WBC (×10^3^/µL)	8.9 (3.20)*	9.1 (3.40)*	<0.001^†^
Neutrophils (%)	66.5 ± 11.5	67.1 ± 11.5	0.205
Lymphocytes (%)	22.1 (7.7)*	21.0 (8.3)*	<0.001^†^
Monocytes (%)	6.8 ± 2.9	6.5 ± 2.4	0.014
Eosinophils (%)	2.1 (2.4)*	2.0 (2.7)*	0.145^†^
Basophils (%)	0.4 (0.30)*	0.4 (0.2)*	0.700^†^
RBC (millions/µL)	4.3 ± 0.6	4.5 ± 0.6	<0.001
HCT (%)	33.0 ± 3.5	38.6 ± 3.6	<0.001
MCV (fL)	78.4 ± 9.9	85.7 ± 8.9	<0.001
MCH (pg)	23.4 ± 4.4	26.6 ± 4.3	<0.001
MCHC (g/dL)	29.6 ± 2.2	30.7 ± 2.1	<0.001
RDW-CV (%)	16.6 ± 3.3	15.1 ± 2.4	<0.001
Platelets (×10^3^/µL)	264.2 (98.0)*	247.0 (99.0)*	<0.001^†^
Reticulocytes (%)	0.8 (0.3)*	0.7 (0.3)*	<0.001^†^
HbA2 (%)	2.9 ± 0.6 (*n* = 1,481)	2.9 ± 0.4 (*n* = 83)	0.67
HbF (%)	0.2 (0.3) (*n* = 1,479)	0.2 (0.2) (*n* = 81)	0.241
Iron (µg/dL)	61.0 (56.0)*	86.0 (52.0)*	<0.001^†^
Magnesium (mg/dL)	1.8 ± 0.1	1.8 ± 0.2	0.001
CRP (mg/L)	5.8 (11.5)*	6.5 (12.2)*	0.021^†^
Albumin (g/dL)	3.7 ± 0.9	3.8 ± 0.4	<0.001
Ferritin (ng/mL)	11.2 (13.5)*	17.3 (14.6)*	<0.001^†^
Vitamin B12 (pg/mL)	212.0 (1.2)*	222.0 (153.2)*	0.012^†^
Vitamin D (ng/mL)	24.3 (12.9)*	24.5 (12.8)*	0.421^†^
Transferrin (mg/dL)	384.4 ± 89.0	351.3 ± 69.9	<0.001
Transferrin Saturation (%)	13.0 (14.7)*	20.1 (13.7)	<0.001
TIBC (µg/dL)	475.4 ± 85.6	438.6 ± 74.7	<0.001
Folate (ng/mL)	7.4 (11.4)*	11.2 (12.0)*	<0.001^†^
Prealbumin (mg/dL)	18.4 ± 4.4	19.9 ± 4.7	<0.001

*P*-value calculated using independent *t*-test, *IQR, and ^†^Mann–Whitney *U*-test.

### 3.7 Association of laboratory markers with anemia

Low iron (aOR 8.23; 95% CI: 5.87–11.54; *p* < 0.001), ferritin (aOR 9.72; 95% CI: 6.16–15.34; *p* < 0.001), folate (aOR 3.13; 95% CI: 2.30–4.26; *p* < 0.001), albumin (aOR 1.57; 95% CI: 1.31–1.89; *p* < 0.001), and prealbumin (aOR 1.55; 95% CI: 1.33–1.81; *p* < 0.001) levels were significantly associated with anemia. Elevated transferrin (aOR 2.05; 95% CI: 1.76–2.39; *p* < 0.001) and TIBC (aOR 2.41; 95% CI: 2.02–2.88; *p* < 0.001) were also associated with anemia. Vitamin B12 and vitamin D were not significantly associated (Table 7).

**Table 7:** Association of various laboratory parameters with prevalence of anemia in pregnant women.

Parameter	Category	Anemic (n, %)	Non-anemic (*n*, %)	aOR (95% CI)	*p*-value
Iron (μg/dL)	Normal (ref)	1,252 (57.9)	910 (42.1)	1.00	**<0** **.** **001**
	Low	453 (91.9)	40 (8.1)	**8.23 (5** **.** **87–11.54)**	**<0** **.** **001**
Ferritin (ng/mL)	Normal (ref)	1,437 (59.9)	962 (40.1)	1.00	**<0** **.** **001**
	Low	316 (93.8)	21 (6.2)	**9.72 (6** **.** **16–15.34)**	**<0** **.** **001**
Transferrin (mg/dL)	Normal (ref)	900 (57.3)	671 (42.7)	1.00	**<0** **.** **001**
	High	876 (73.2)	321 (26.8)	**2.05 (1** **.** **76–2.39)**	**<0** **.** **001**
TIBC (μg/dL)	Normal (ref)	1,082 (57.7)	792 (42.3)	1.00	**<0** **.** **001**
	High	713 (77.6)	206 (22.4)	**2.41 (2** **.** **02–2.88)**	**<0** **.** **001**
Albumin (g/dL)	Normal (ref)	1,290 (61.7)	801 (38.3)	1.00	**<0** **.** **001**
	Low	507 (71.5)	202 (28.5)	**1.57 (1** **.** **31–1.89)**	**<0** **.** **001**
Prealbumin (mg/dL)	Normal (ref)	987 (60.2)	652 (39.8)	1.00	**<0** **.** **001**
	Low	808 (70.1)	345 (29.9)	**1.55 (1** **.** **33–1.81)**	**<0** **.** **001**
Folate (ng/mL)	Normal (ref)	1,227 (62.2)	746 (37.8)	1.00	**<0** **.** **001**
	Low	280 (83.8)	54 (16.2)	**3.13 (2** **.** **30–4.26)**	**<0** **.** **001**
Vitamin B12 (pg/mL)	Normal (ref)	692 (62.3)	420 (37.7)	1.00	0.118
	Low	1,086 (65.7)	567 (34.3)	1.16 (0.96–1.39)	0.130
Vitamin D (ng/mL)	Sufficient (ref)	483 (62.1)	295 (37.9)	1.00	0.506
	Deficient	589 (64.8)	322 (35.2)	1.12 (0.93–1.35)	0.230
CRP (mg/L)	Normal (ref)	1,176 (64.9)	638 (35.1)	1.00	0.352
	High	625 (63.1)	366 (36.9)	0.92 (0.79–1.08)	0.310

Bold text represents significant *p*-value.

### 3.8 Multivariable model for independent predictors

A multivariable logistic regression analysis was performed to identify independent predictors of anemia. The model demonstrated a good fit, as indicated by a non-significant Hosmer-Lemeshow test (*χ*^2^ = 9.24, df = 8, *p* = 0.322). Furthermore, all Variance Inflation Factor (VIF) values were below 2.0, indicating no concerning multicollinearity. The analysis revealed five independent factors significantly linked to anemia in pregnant women. Among them, a low red blood cell (RBC) count notably elevated the risk of developing anemia. (aOR = 0.26; 95% CI: 0.22–0.31; *p* < 0.001). Higher red cell distribution width (RDW-CV) was positively associated with anemia (aOR = 1.39; 95% CI: 1.33–1.46; *p* < 0.001). Low serum prealbumin (aOR = 0.92; 95% CI: 0.90–0.94; *p* < 0.001), low folate (aOR = 0.97; 95% CI: 0.96–0.98; *p* < 0.001), and low ferritin levels (aOR = 0.99; 95% CI: 0.996–0.999; *p* < 0.001) were also identified as independent variables foe anemia prevalence (Table 8).

**Table 8:** Multivariable logistic regression analysis for identification of independent predictors of anemia.

Predictor	β (SE)	Adjusted OR	95% CI	*p*-value	VIF
Intercept	3.38 (0.48)	29.41	11.42–75.72	<0.001	–
RBC (×10⁶/μL)	−1.35 (0.09)	**0** **.** **26**	0.22–0.31	<0.001	1.21
RDW-CV (%)	0.33 (0.02)	**1** **.** **39**	1.33–1.46	<0.001	1.21
Prealbumin (mg/dL)	−0.08 (0.01)	**0** **.** **92**	0.90–0.94	<0.001	1.02
Folate (ng/mL)	−0.03 (0.01)	**0** **.** **97**	0.96–0.98	<0.001	1.01
Ferritin (ng/mL)	−0.003 (0.001)	**0** **.** **99**	0.996–0.999	<0.001	1.01

Model Fit Statistics: *χ*^2^ = 581 (df = 5, *p* < 0.001), McFadden's R^2^ = 0.159, AIC = 3,087, BIC = 3,123, AUC = 0.763 (95% CI: 0.747–0.779), Sensitivity = 86.7%, Specificity = 48.3%, Classification Accuracy = 72.9%.

## 4 Discussion

This study presents a comprehensive and regionally representative analysis of anemia among pregnant women in Gujarat, aiming to detect its multifactorial etiology through integrated sociodemographic, nutritional, clinical, biochemical, and hematological assessments. Conducted across ten diverse districts and involving 2,805 pregnant women, it offers one of the most detailed district-level assessments of anemia in India to date. The persistent high burden of anemia, despite ongoing public health interventions, underscores the urgency of such granular analyses. In the context of the Government of India's Anemia Mukt Bharat initiative, identifying localized and modifiable risk factors is essential for refining intervention strategies.

The prevalence of anemia in pregnant women reported in this study (64.2%) exceeds both global and National averages. According to WHO estimates, approximately 40% of pregnant women worldwide are anemic, with the highest prevalence recorded in sub-Saharan Africa (57%) and South Asia (52%). (1) Comparable studies from Bangladesh (46.8%), Nepal (42%), and Ethiopia (56.8%) demonstrate a similar magnitude of the problem, emphasizing that anemia remains a leading cause of adverse maternal and perinatal outcomes globally (2, 20, 21). The predominance of mild anemia (82.1%) in our study aligns with global findings, where mild-to-moderate anemia constitutes the majority of cases in low- and middle-income countries (LMICs). However, this overall pattern of mild anemia masks substantial variation in clinical and biochemical characteristics (22), indicating that a uniform public health approach may not be sufficient to address the diverse underlying causes. Notably, no district reported a prevalence below 50%, indicating anemia's endemic nature. Tribal-dominated districts exhibited the highest burden, pointing to systemic determinants such as poverty, healthcare inaccessibility, and geographic remoteness (23). Similar findings have been reported in tribal populations across India, where logistical barriers limit antenatal care and nutrition services (24, 25).

Among the most influential sociodemographic predictors were tribal status, illiteracy, poor housing (kutcha structures), and poverty. Tribal women had 2.21 times the odds of anemia compared to urban counterparts, consistent with data from the International Institute of Population Sciences (IIPS) (23). Illiterate women showed a 74.7% prevalence, affirming the established link between maternal education, dietary behavior, and health service utilization (26–28). Nutritional vulnerability, reflected by lower BMI and body weight, emerged as a key risk factor, supported by significantly lower albumin and prealbumin levels in anemic women. The latter parameters—reduced in 28% and 37% respectively—biochemically confirmed the role of protein-energy malnutrition (29, 30).

The analysis of healthcare utilization patterns revealed key areas for strengthening service delivery. Differences in anemia prevalence across delivery settings may reflect variations in the timing and continuity of antenatal care services, including screening protocols and adherence to supplementation guidelines. These results underscore the need to strengthen and standardize government-run antenatal initiatives to guarantee that all pregnant women—irrespective of their geographic location or the type of health facility they visit—have access to complete and consistent maternal care (31–33). Notably, anemia prevalence was highest during the second trimester, which may be attributed to physiological plasma volume expansion (34, 35) and increased iron demands during this stage of fetal development (36)— a phenomenon consistently reported across maternal nutrition and reproductive health studies.

At the biological level, this study adds new evidence by integrating hematological and biochemical markers to understand population-level anemia risk. Elevated red cell distribution width (RDW-CV) and lower mean corpuscular volume (MCV), both identified as key predictors in multivariate analysis, reflect disruptions in red blood cell structure and hemoglobin production (37, 38)—patterns often seen in conditions linked to micronutrient deficiencies and hemoglobin variants. The high odds ratio for low hematocrit (aOR = 58.9) emphasizes its diagnostic value in identifying moderate-to-severe cases. Low RBC count, which emerged as the most powerful inverse predictor (aOR = 0.26), further strengthens the argument that red cell production and morphology are central to anemia pathogenesis in this setting (39).

The findings reveal that just 17.2% of anemia cases in pregnancy were due to absolute iron deficiency, questioning the prevailing belief that iron deficiency is the predominant cause of anemia among Indian pregnant women. Folate (83.8%) and vitamin B12 (60.3%) deficiencies were highly prevalent, particularly in vegetarian populations—a pattern aligned with prior regional studies (40–42). Folate's role in erythropoiesis and DNA synthesis underscores its centrality in anemia pathophysiology (43). This biochemical heterogeneity reinforces WHO's call for multi-micronutrient supplementation rather than iron-only approaches in pregnancy (44, 45).

Moreover, protein status, often overlooked in anemia assessments, emerged as a significant determinant. Lower prealbumin levels (aOR = 1.55) and serum albumin (aOR = 1.57) were independently associated with anemia. These markers reflect not just dietary inadequacy but also systemic inflammation and hepatic synthetic dysfunction, which have been implicated in functional iron deficiency and impaired hemoglobin synthesis (46, 47). These findings suggest that addressing macronutrient deficits may be as crucial as correcting iron insufficiency (48).

Inflammation, as assessed via CRP levels, was elevated in 34.7% of anemic cases, particularly in Mehsana and Dahod. CRP elevation suggests inflammation-induced hepcidin upregulation, which inhibits iron absorption and promotes sequestration, even when iron stores (e.g., ferritin) appear adequate (49, 50). This mechanism likely explains anemia in many cases without absolute iron deficiency. Additionally, higher CRP levels among overweight and obese women in the cohort support existing evidence linking chronic inflammation from adiposity to impaired iron metabolism and anemia of chronic disease (51).

District-level analyses revealed geographic heterogeneity in underlying causes. Mehsana and Sabarkantha had higher iron deficiency rates, while B12 and protein deficiencies dominated in Dahod, Anand, and Navsari. This spatial variation aligns with NFHS-5 zonal trends but adds biochemical granularity, enabling more precise targeting of interventions. The iron absorption dynamics influenced by inflammation and metabolism (e.g., hepcidin modulation) warrant integration into public health anemia management (52).

Hemoglobinopathies, assessed via HPLC, had a prevalence of 11.1%, consistent with prior findings from tribal-dense areas of Gujarat (53, 54). While not modifiable through nutrition, awareness, and screening of hemoglobinopathies are essential components of a comprehensive anemia control strategy.

Our final multivariable logistic regression identified five independent predictors—low RBC count, high RDW-CV, low prealbumin, low folate, and low ferritin—forming an efficient yet explanatory model with good discrimination (AUC = 0.763). These parameters are easily obtainable in most clinical settings and may help refine screening and risk stratification protocols in antenatal care. Moreover, their biological plausibility underlines their potential for informing targeted interventions. For example, targeting protein malnutrition alongside folate and iron supplementation could potentially yield better hemoglobin gains than iron alone (48)—a hypothesis worthy of future trials.

This study demonstrates several methodological strengths. The large sample size and representation across ten diverse districts enhance generalizability. The integration of clinical, biochemical, and hematological data permits differentiation between nutritional, inflammatory, and genetic causes. Only 17.2% of anemic cases had absolute iron deficiency, while 34.7% had high CRP—highlighting the limitation of hemoglobin as a standalone metric. The inclusion of variables like RDW-CV, RBC count, and micronutrient markers in multivariable modeling allowed for a nuanced understanding of anemia's multifactorial etiology. Disaggregated data further revealed localized drivers, such as high folate deficiency in Sabarkantha and B12 deficits in Dahod, reinforcing the need for region-specific programming.

These findings should be interpreted with caution due to certain limitations. Primarily, the cross-sectional design of the study restricts our ability to infer causal relationships between the identified factors and anemia outcomes. Second, our reliance on self-reported data for dietary patterns, iron-folic acid supplement usage, and hygiene practices may introduce potential biases, as participants might inaccurately recall information or provide socially desirable responses. These methodological constraints suggest the need for caution when drawing definitive conclusions from our results. Though multiple biomarkers were included, others such as hepcidin or interleukins could have improved mechanistic insights. Lastly, the absence of longitudinal follow-up limits understanding of intervention impact and anemia progression over time.

### 4.1 Policy and programmatic implications

**Figure 2:**
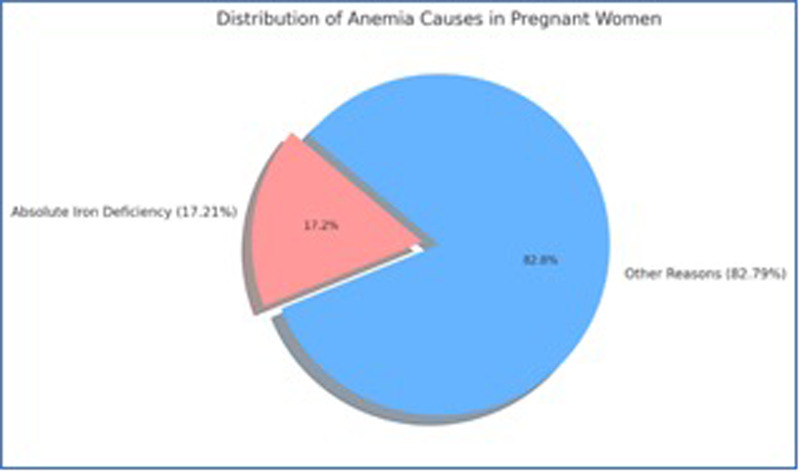
Distribution of anemia causes in pregnant women.

The findings of this study carry important implications for national public health programs such as the Anemia Mukt Bharat (AMB) strategy and the Reproductive, Maternal, Newborn, Child, and Adolescent Health (RMNCH + A) framework. The evidence showing that only 17.2% of anemia cases were due to absolute iron deficiency (Figure 2) calls for a recalibration of existing interventions, which predominantly emphasize iron and folic acid supplementation. Strengthening AMB's implementation can be achieved by incorporating biochemical screening for folate, vitamin B12, and protein status within routine antenatal check-ups. This would facilitate differential diagnosis and enable tailored supplementation rather than a one-size-fits-all approach.

Additionally, the study underscores the importance of intersectoral coordination between health, nutrition, and food security programs. Collaboration with the Integrated Child Development Services (ICDS) and the Public Distribution System (PDS) could enhance dietary diversity and access to fortified food products. Under RMNCH + A, reinforcing antenatal care continuity, ensuring early registration, and integrating counseling on balanced diets and protein intake at every contact point could significantly improve maternal nutrition outcomes.

At the system level, capacity building of frontline health workers (ASHAs, ANMs) is essential to improve screening accuracy, supplement adherence, and community follow-up. Incorporating digital monitoring tools under the Health Management Information System (HMIS) could also strengthen supply chain accountability and ensure timely availability of IFA and multi-micronutrient supplements at the point of care.

### 4.2 Barriers and practical considerations

Despite multiple policy efforts, several barriers continue to impede effective anemia control. Poor compliance with iron and folic acid supplementation—often due to side effects, misconceptions, and irregular supply—remains a major challenge. Food insecurity and limited dietary diversity, particularly in tribal and low-income households, further exacerbate micronutrient deficiencies. In addition, regional inequalities in access to quality antenatal services, laboratory diagnostics, and nutrition counseling perpetuate disparities in anemia prevalence across districts.

To overcome these barriers, community-based behavior change communication (BCC) should be intensified, focusing on the importance of continuous supplementation and nutrient-rich diets. Introducing locally acceptable fortified food options, promoting kitchen gardens, and engaging Self-Help Groups (SHGs) can help address food insecurity at the grassroots level. Moreover, micro-planning at the district and block level—using local surveillance data to prioritize high-burden areas—can ensure context-specific allocation of resources and targeted interventions.

### 4.3 Future research directions

Future research should move beyond cross-sectional analyses to longitudinal and cohort-based designs that can elucidate the causal pathways underlying the persistence of anemia among pregnant women. Integrating environmental, socio-cultural, and health system variables into such studies will be essential to understand the broader determinants influencing anemia trends across diverse settings. In particular, future studies should explore how factors such as water quality, sanitation, agricultural diversity, and climate-related food insecurity impact maternal micronutrient status.

Furthermore, examining behavioral determinants—including health-seeking behavior, cultural beliefs surrounding diet during pregnancy, and adherence to iron and folic acid supplementation—can provide deeper insight into the barriers to program effectiveness. Mixed-method approaches combining quantitative data with qualitative community perspectives would be valuable for identifying context-specific drivers of anemia and improving intervention design.

Implementation research should also focus on assessing the real-world effectiveness and cost-efficiency of ongoing interventions such as iron–folic acid supplementation, multi-micronutrient fortification, and protein-rich dietary diversification within public health programs. Evaluating the health system's readiness, supply chain functionality, and frontline worker capacity to deliver these interventions can generate actionable evidence for program strengthening. Collectively, these research directions can support the refinement of national initiatives such as Anemia Mukt Bharat and RMNCH + A, ensuring that policies are more adaptive to regional needs and grounded in implementation evidence.

## 5 Conclusion

Anemia remains highly prevalent among pregnant women in Gujarat, with significant geographic and sociodemographic variation. While iron deficiency contributes, other factors—including folate and protein deficiency, inflammation, and hemoglobinopathies—emerged as key determinants. The study underscores the need for integrated, evidence-based, and region-specific strategies to reduce the anemia burden effectively.

## Data Availability

The raw data supporting the conclusions of this article will be made available by the authors, without undue reservation.
